# Automated tissue dissociation for the establishment of human intestinal organoids

**DOI:** 10.1038/s41598-025-03905-9

**Published:** 2025-06-05

**Authors:** Woo Jin Yang, Eva Blahusova, Komal Nayak, Róisín M. Owens, Matthias Zilbauer

**Affiliations:** 1 Cambridge Stem Cell Institute, University of Cambridge, Cambridge, UK; 2 Department of Chemical Engineering and Biotechnology, University of Cambridge, Cambridge, UK

**Keywords:** Human intestinal organoids, Tissue dissociation, Automation, Intestinal stem cells, Gastrointestinal models, Tissue culture

## Abstract

Human intestinal Organoids (HIOs) may be set up from primary human tissue following dissociation, release, and culture of intestinal LGR5 + stem cells. Here, we characterise the use of a semi-automated mechanical tissue dissociation technique, which demonstrates an expedited workflow and streamlined protocol, standardising key aspects of the process while consistently maintaining a high level of cultured organoid viability.

**Supplementary Information:**

The online version contains supplementary material available at 10.1038/s41598-025-03905-9.

## Introduction

Human intestinal Organoids (HIOs)^1^ represent an intricate 3D model able to recapitulate the crypt-villus-like architecture and cellular composition of the in vivo intestinal cellular mise-en-scene and can recreate physiological and cellular characteristics to a greater fidelity than offered by cell lines and other monocultures. However, HIO establishment necessitates the isolation and culture of intestinal crypts with LGR5 + stem cells from primary tissue. To facilitate isolation and release of these crypts, primary tissue is conventionally processed via EDTA^2^ or enzyme-based^3^ dissociation, leaving sample tissue subject to extended exposure to cytotoxic dissociative materials. Moreover, conventional methods require the manual disruption of crypts through repeated pipetting^2^, which may introduce variability between operators.

**Fig. 1:**
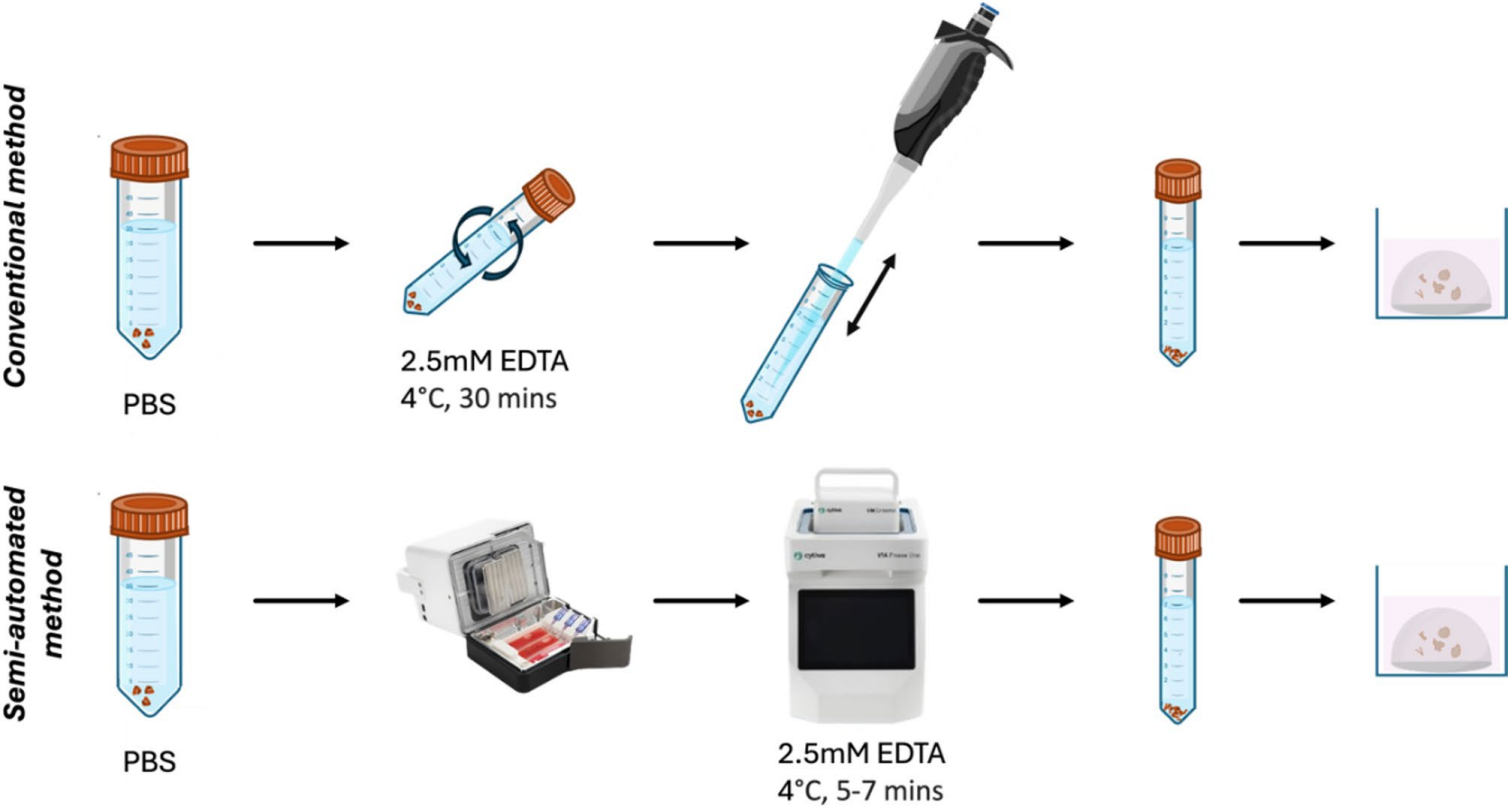
Illustrated workflow of the conventional and semi-automated protocols for tissue dissociation.

Here, we report a semi-automated protocol for primary tissue dissociation using EDTA, successfully establishing organoid cultures from fresh and cryopreserved samples, and demonstrating improved efficiency through a novel semi-automated platform (Fig. 1). This protocol reduces time taken for tissue processing—thus reducing prolonged exposure to dissociative reagents—and provides a standardised organoid establishment workflow with improved consistency.

## Results

**Fig. 2:**
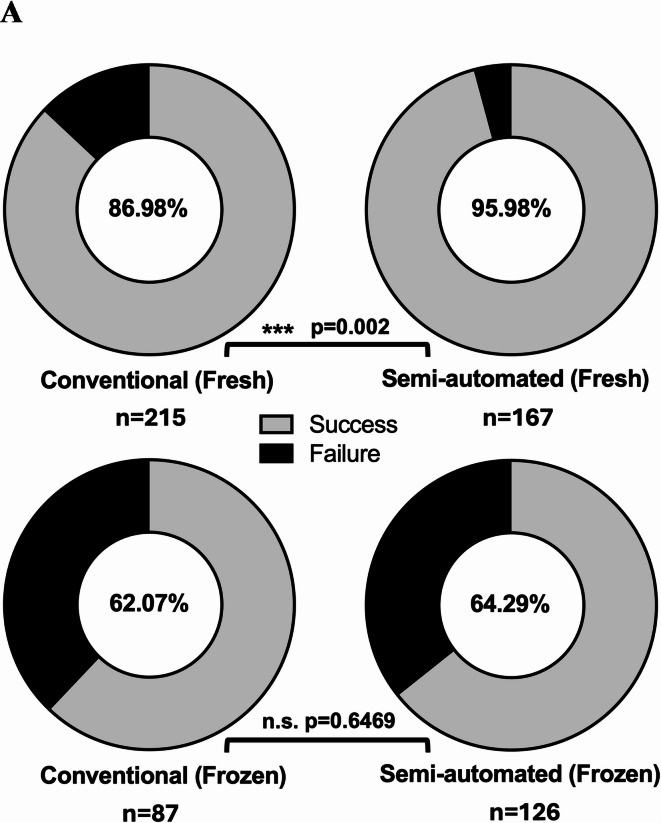

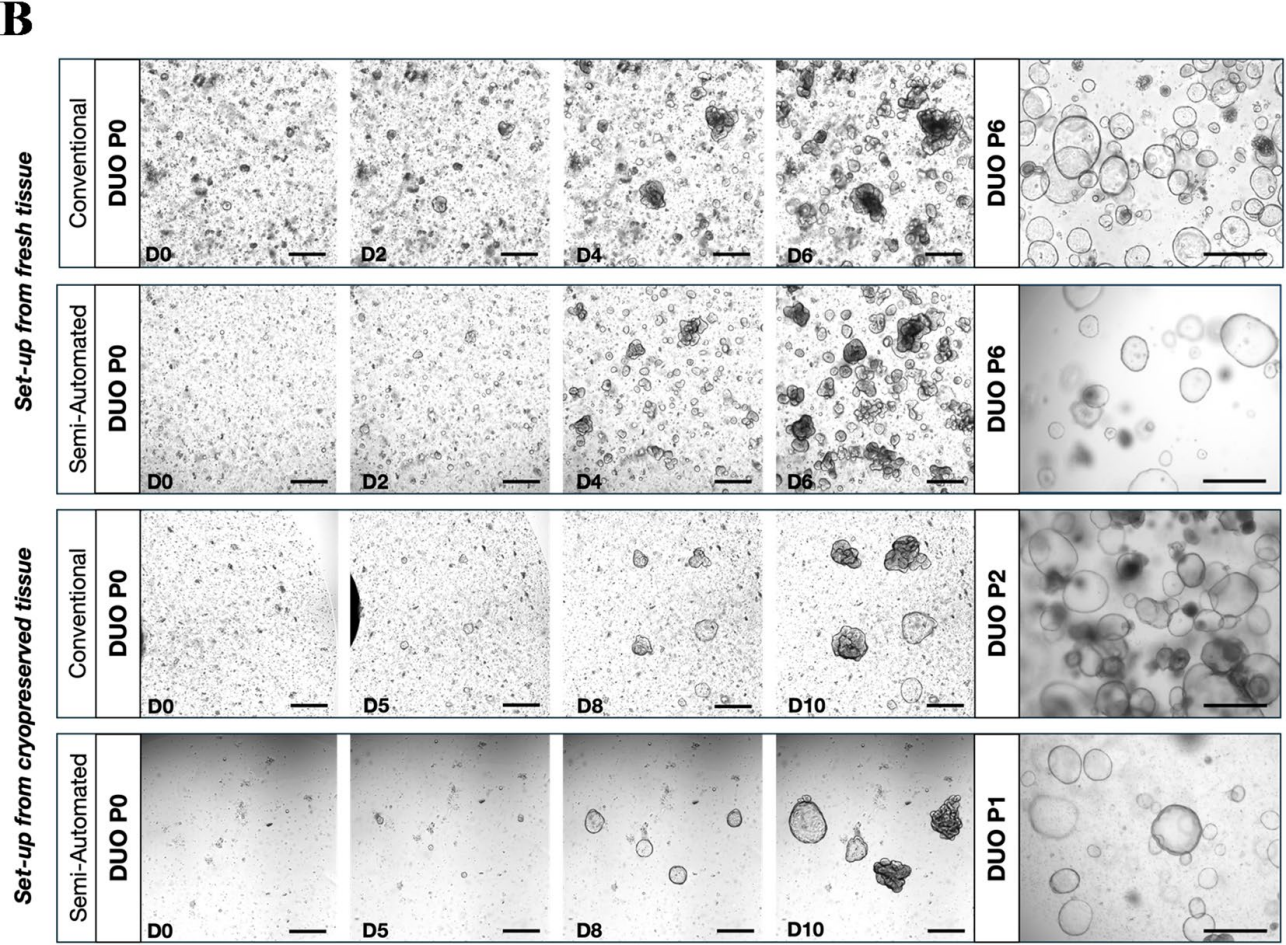
(A) Rate of successful organoid derivation from the different methodologies. Success was defined by the visible presence or the lack thereof of organoids at P0. Significance as calculated by a binomial test of expected conventional set up efficacy. (B) Representative images of duodenal organoids cultured following isolation via Conventional and Semi-automated dissociation. Scale bars: (Left panels) 400 μm for P0, (Right panels) 750 μm.

Human intestinal mucosal biopsies were processed to isolate crypts for organoid culture over the span of 2 years, where fresh tissue dissociated via the semi-automated system demonstrated an improved rate of successful organoid derivation (Fig. 2A).

For direct comparison, organoids were established from patient-matched mucosal biopsies dissociated via either the conventional or semi-automated method. No significant visual morphological disparities were observed between the two organoid cultures, both on initial culture (Fig. 2B, Left panels), as well as during later passages (Fig. 2B, Right panels). This is consistent with organoids set up from frozen biopsies, suggesting that the semi-automated dissociation is suitable for extraction from cryopreserved equally as fresh tissue samples.

**Fig. 3:**
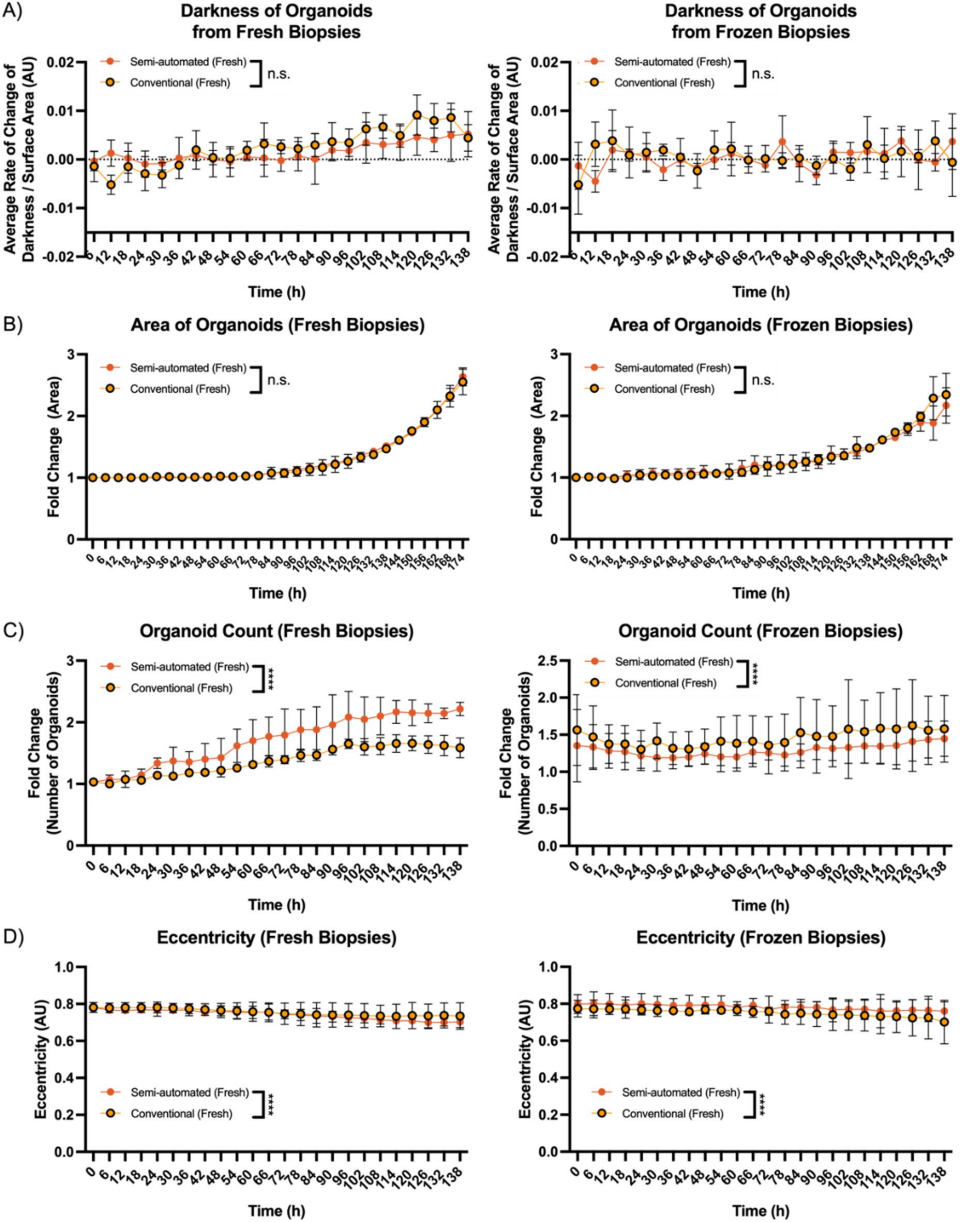
Incucyte data for growth curves of average modified darkness, Area, Number, and Eccentricity of organoids. Data for several Incucyte parameters detailing growth kinetics of duodenal organoids from patient-matched conventional and semi-automated dissociated tissue, *n* = 3. 3 A) Modified organoid darkness 3B) Area of organoids 3 C) Number of organoids produced from a single biopsy 3D) Average measure of eccentricity. Statistical comparisons were carried out with one-way repeated measures Analysis of Variance (ANOVA). ****<0.0001. Error bars represent ± SD.

Analysis of the organoid growth kinetics suggests that there is no significant difference in organoid growth and recovery (Fig. 3B, Area) and in modified darkness as a function of surface area which serves as a metric of cell shedding and viability^4^ (Fig. 3A). However, isolation efficacy from biopsies (Fig. 3C, number of organoids per well), and budding characteristics (Fig. 3D) suggest that the semi-automated dissociation may be better suited for fresh tissue, and the conventional method for frozen biopsies. This difference may arise from the reduced physical tissue integrity of frozen tissue which may leave it more susceptible to physical processing, while the thorough nature of the semi-automated digestion provides greater yield for fresh biopsies. Overall, we suggest that there is no significant difference in organoid growth efficacies and dynamics between the conventional and semi-automated dissociation protocols, although a preference for semi-automated digestion may be made for more efficacious crypt isolation from fresh biopsies (and vice versa).

**Fig. 4:**
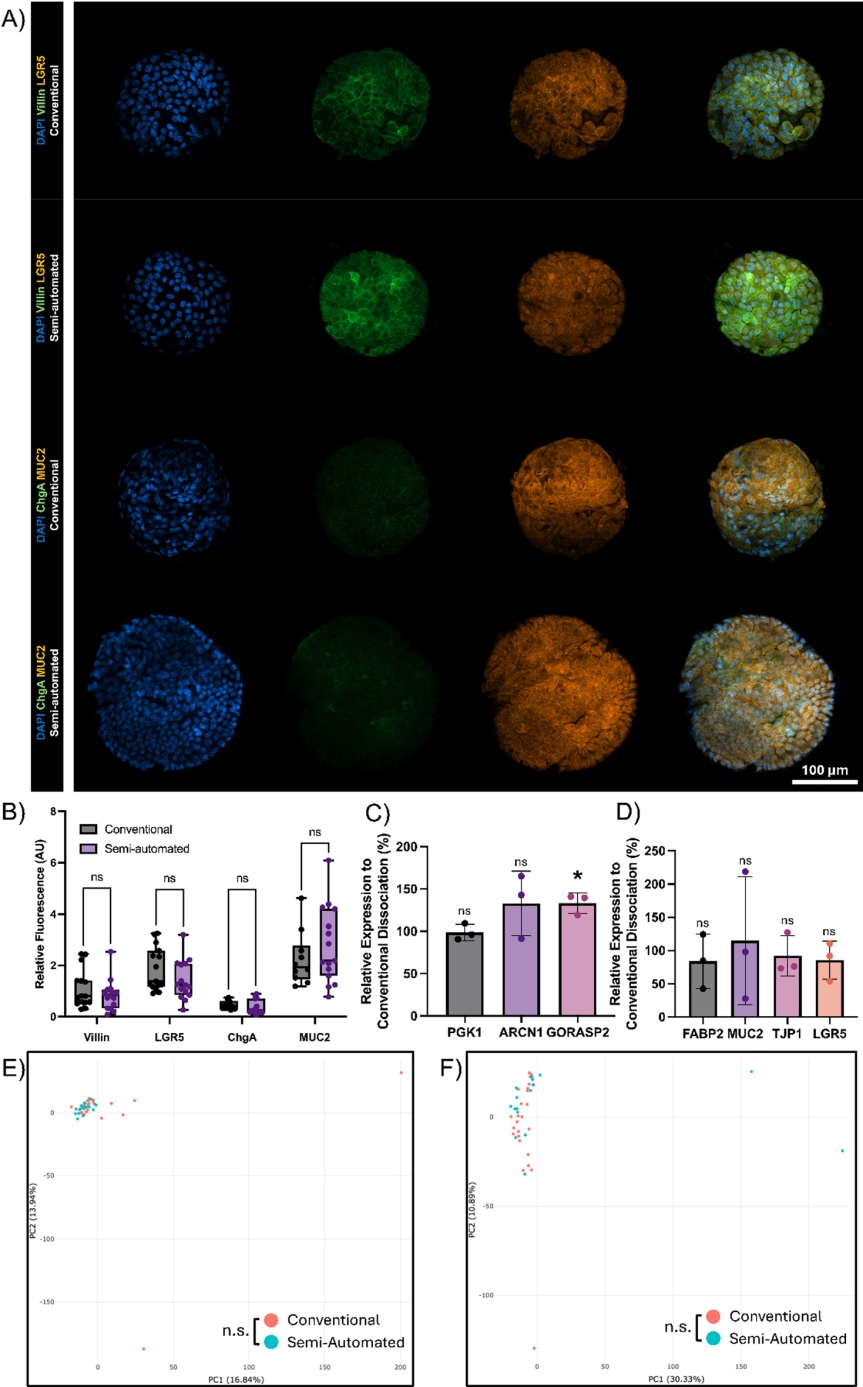
Validation of cell type markers and epigenetic profile. 4 A**)** Immunostaining comparison for the different dissociation methods. Duodenal organoids established from fresh tissue. Markers of LGR5, ChgA, MUC2 used for Stem cells, Enteroendocrine, and Goblet cells respectively on representative organoids. 4B) Quantification of duodenal organoid immunostaining signal intensity relative to DAPI. *n* = 3 biological replicates, *n* ≥ 3 technical replicates. 4 C) Relative expression of stress genes of P1 Organoids (derived from fresh biopsies) as a marker of organoid stress, *n* = 3, student’s t-test. 4D) Relative expression of cell marker genes (FABP2 – Enterocytes, MUC2 – Goblet cells, LGR5 – Stem cells) and tight junction function (TJP1), *n* = 3, student’s t-test. Relative expressions calculated as a ratio of (Semi-automated : Conventional). 4E-F) PCA plot of highly variable CpGs for (E) Terminal Ileum and (F) Sigmoid Colon-derived organoids from *n* ≥ 16 different patients. Significance calculated between the first 6 principal components via MANOVA. *p* = 0.3412 (E), *p* = 0.3186 (F). Error bars represent ± SD. *<0.05.

Furthermore, we show that there is no visual difference in the proportion of cell types in the organoids established from the disparate methods. Populations of LGR5 + stem cells and MUC2 + Goblet cells, (Fig. 4A-B) as well as relative expression levels of cell markers^1,5^ and function^6^ (Fig. 4D) remain in similar proportions in organoids derived from conventional or semi-automated dissociation. Moreover, ChgA + Enteroendocrine cells remain absent in both conditions under expansion medium, thus demonstrating the absence of aberrant differentiation (Fig. 4A), and organoids from semi-automated dissociation demonstrate the standard apical-in phenotype (Supplementary Fig. 1). In addition, there is no change in the expression of oxidative stress markers PGK1^7^ and ARCN1^8^. While GORASP2^9,10^ is slightly elevated (*p* = 0.0414) in the semi-automated dissociation, we may consider this difference relatively unimportant alone in consideration of PGK1, ARCN1 (Fig. 4C), and growth kinetics data (Fig. 2). Moreover, we demonstrate a non-significant difference in the methylation profile of the differentially dissociated organoids (Fig. 4E-F), showing that the established organoids have comparable phenotype across methylation, RNA expression, and at the protein-level.

## Discussion

Altogether, we may safely ascertain that there are no significant changes in the growth kinetics of organoids set up from the two different dissociation methods, as characterised by live organoid imaging and growth kinetic analysis, qPCR, and immunostaining. We therefore conclude that the semi-automated mechanical dissociation method is able to sufficiently and viably dissociate primary human tissue to more efficaciously establish organoids indistinguishable from those constituted from conventional methods with its faster workflow and streamlined process ensuring key steps are followed consistently. We suggest that this gives rise to the observed improved success rate (Fig. 2A) and isolation efficacy (Fig. 3C) for fresh tissue, as the nature of a semi-automated system presents a platform which helps reduce user-based variability, thus methodically providing greater cell yield. This improvement is not significant for frozen tissue due to the loss of viability during cryopreservation which most likely serves as the main limiting factor although further improvements in cryopreservation may help ameliorate this problem.

### Troubleshooting

Various issues may be encountered during the use of this protocol - please ensure that all reagents and tissues are stored as appropriate. Common problems and solutions are outlined in Table 1.

**Table 1:** Troubleshooting common issues encountered during tissue dissociation for intestinal crypt isolation via semi-automated system.

Problem	Solution
Biopsy adhesion to Via Extractor Sachet / pipette tips	Pre-coat plastic containers and equipment with 0.1% BSA prior to addition of biopsies
Organoids fail to grow from the dissociation mixture	1. Ensure crypts are fully released and tissue is not under-dissociated (but avoid over dissociation)
	2. Minimise exposure to EDTA solution
	3. Maintain dissociation temperature at 4 °C
Organoids show impaired growth	Adjust dissociation parameters to balance cell yield and viability, ensuring crypts (not single cells) are isolated

## Methods

### Organoid culture

#### Paediatric mucosal biopsies

Biopsies were collected from children under 16 years of age undergoing diagnostic endoscopy, performed by consultant paediatric gastroenterologists at Addenbrooke’s Hospital (Cambridge University Hospitals NHS Foundation Trust, UK). One to three forceps biopsies were taken from each of the duodenum (Duo), terminal ileum (TI), sigmoid colon (SC). Children with no signs of underlying gastrointestinal disease and macroscopically and histologically normal mucosa were classified as healthy controls.

Fresh tissue refers to tissue processed on the day of collection; frozen tissue refers to tissue cryopreserved for > 3 weeks.

Human HIOs from mucosal tissue were set up according to a protocol adapted from Sato^16^. Mucosal biopsies were washed three times with cold, sterile phosphate buffered saline (PBS) to isolate microbiota. Biopsies were either:

1. Conventional: Incubated in 2.5 mM EDTA in PBS for 30 min at 4 °C on a roller-mixer. After 3 washes with cold PBS to remove residual EDTA, intestinal crypts were released into PBS by pipetting with a P1000 pipette. The crypt suspension was collected, and this process was repeated three times.
2. Semi-automated (*Cytiva Via Extractor*): Placed in 0.1% BSA-coated pouches with 5 ml 2.5mM EDTA, sealed in a sterile environment, and dissociated via the Via Extractor to release crypts from tissue (for HIOs). Runs were carried out at 4 °C, and time and dissociation intensity were used under optimised conditions (150 rpm, 7 min for fresh tissue, 5 min for cryopreserved tissue) for crypt isolation.

Crypt suspensions were centrifuged at 800 x g for 5 min and resuspended in Matrigel matrix (Corning). Around 100 crypts were seeded per well in 20 µl of Matrigel on a pre-warmed 48-well plate and incubated at 37 °C for 5–10 min to allow gel polymerisation. Plates were incubated in an inverted position to stop organoids settling to the well bottom. Complete expansion medium for human intestinal epithelial organoid culture (WENRAS – composition provided in Table 2) supplemented with 10 µM Rho-kinase inhibitor Y-27,632 (Y, Sigma-Aldrich) was added on top of Matrigel domes. Cultures were then kept at 37 °C, 7% CO2. Complete culture medium was freshly made from stock solutions stored at -20 °C and used for up to one week. Medium including Y (CMY) was replenished the next day. Cultures were maintained with fresh medium (CM) every two to three days and passaged every seven to ten days.

**Table 2:** Complete expansion medium for human intestinal epithelial culture (WENRAS).

Basal Organoid Medium = ADF+++ Products from Gibco by Thermo Fisher Scientific	Final concentration
Advanced DMEM/F12 (ADF)	1x
GlutaMax	2 mM
HEPES buffer	10 mM
Penicillin/Streptomycin	0.5 U/ml

Complete medium (CM)	Final concentration
ADF+++ (see above)	76.3% (vol/vol)
WNT-Fc surrogate protein (Thermo Fisher Scientific)	0.2nM
R-spondin-1 conditioned medium	20% (vol/vol)
Primocin (Invivogen San Diego, CA, USA)	500 µg/mL
B-27^®^ Supplement (Invitrogen, Carlsbad, CA, USA)	1x
Nicotinamide (Sigma, St. Louis, MO, USA)	10 mM
N-Acetylcysteine (Sigma, St. Louis, MO, USA)	1.25 mM
A3801 (Tocris, Bristol, UK)	500 nM
SB202190 (Sigma, St. Louis, MO, USA)	10 µM
Murine EGF (Invitrogen, Carlsbad, CA, USA)	50 ng/mL
Murine Noggin (Peprotech, Rocky Hill, NJ, USA)	100 ng/mL

#### Organoid passaging

Human organoids were maintained by splitting in a ratio 1:3 to 1:4 every seven to ten days. Organoids were recovered from Matrigel via mechanical disruption and split with a P1000 pipette into visibly smaller fragments, centrifuged at 800 g for 5 min, resuspended in fresh Matrigel and plated as described above.

#### Imaging

Live organoids were monitored and captured at 6-hour intervals using the Sartorius Incucyte (Organoid ◊ QC scan setting) and were characterised for several parameters using the built-in organoid detection software. Thresholds for organoid detection were kept at a high preference for organoids over the environment to ensure individual detection of organoids to preserve data quality.

Confocal imaging was carried out for organoids (Zeiss LSM 880) with a 10x/20x objective lens and post-processed using Zeiss ZenBlue software.

#### Data post-processing

Data analysis was performed on the four output parameters quantified by the Incucyte: area per organoid, count, eccentricity, and darkness. Time series data were statistically compared using a repeated-measures two-way ANOVA to characterise the effects of time (to demonstrate statistically significant growth) as well as any condition-specific differences.

High variability encountered in organoid area because of false detection of tissue debris as organoids was accounted for by treating the lowest average area as the starting point for data. To account for this, all datapoints for average area were normalised to the global minima, after which previous datapoints were adjusted to the global minima, then were translationally adjusted for time (i.e., point at which the minima began were set to the same relative timepoint), then were averaged across different samples from different patients. This was done to bypass patient-by-patient variability as well as differences in initial growth kinetics from a different amount of starting material. As the measure of average darkness is directly correlated with organoid size, we sought to normalise this by estimated organoid surface area^4^.

### DNA/RNA extraction

4 wells of 5-day old organoids were harvested by resuspension and washing in PBS (3x), from which DNA and RNA were extracted using the AllPrep DNA/RNA/Protein Mini Kit (QIAGEN) per the manufacturer’s instructions.

### Reverse transcription and quantitative PCR

RNA was reverse-transcribed and used in quantitative PCR (qPCR) as described previously^11^. Relative expression was calculated using the ΔΔCt method^12^. Primers are provided in Table 3.

**Table 3:** qPCR targets and their respective primers.

Target	Forward primer	Reverse primer
PGK1	TGGACGTTAAAGGGAAGCGG	GCTCATAAGGACTACCGACTTGG
ARCN1	ATTGCCGAGCCTTAGAAGAGA	CCCAGTGCGACAATTTCATCA
GORASP2	AACATTGGAACTGCGAGAGAC	TGCAGAAACGAATGCTCACTC
LGR5	CACCTCCTACCTAGACCTCAGT	CGCAAGACGTAACTCCTCCAG
FABP2	ATGGCGTTTGACAGCACTTG	TCAGTTCCGTCTGCTAGATTGTA
TJP1	CAACATACAGTGACGCTTCACA	CACTATTGACGTTTCCCCACTC
MUC2	GAGGGCAGAACCCGAAACC	GGCGAAGTTGTAGTCGCAGAG

### DNA methylation

Extracted DNA was bisulfite converted with the EZ DNA Methylation-Gold Kit (Zymo Research). Genome-wide DNAm was profiled using the Infinium MethylationEPIC v2.0 Kit. DNAm data was processed using the minfi package and normalised based on control probes on each array using functional normalisation^13^. Samples were removed if they had high average detection p-values (> 0.05) across all probes. Batch effect was removed by using ComBat^14^ and probes were filtered for quality. The CpGs with the top 10% variance were sorted and used to derive a PCA plot for mechanical vs. semi-automated dissociation methods for Terminal Ileum and Sigmoid Colon derived organoids.

### Staining

HIOs were passaged as described above, plated in 48-well culture plates in 20 µl of Matrigel per well, and grown for 5 days. The Matrigel was dissolved in 1 ml PBS, and organoids pelleted down for 3x washing steps to remove debris and matrigel. They were then fixed in 4% formaldehyde solution (Sigma-Aldrich) for 10 min with initial pipetting for the first 5 min with a 2% BSA coated pipette tip to prevent organoid clumping, permeabilised with 0.5% Triton X-100 (Thermo Fisher) in PBS for 5 min and blocked with 1% normal BSA for 30 min at room temperature. Staining was performed by using primary antibodies diluted in 1:100 1% BSA in Eppendorf tubes overnight at 4 °C (200 µl/tube). Following removal of excess primary antibody solution, secondary antibodies diluted 1:450 in 1% BSA (45-minute incubation, room temperature) were added. Samples were then washed with PBS 3x then counterstaining with 1 ug/ml DAPI (Thermo Fisher) in PBS for 5 min at room temperature. HIOs were washed 3 times with PBS between each step. Antibodies and suppliers provided in Table 4.

**Table 4:** Antibodies and suppliers.

Target	Product code	Supplier
ChgA	sc-393,941	Santa Cruz Biotechnology
LGR5	ab261734	ABCAM
Villin	sc-58,897	Santa Cruz Biotechnology
MUC2	ab134119	ABCAM
Secondary Goat Anti-Rabbit IgG H&L (Alexa Fluor^®^ 555)	ab150078	ABCAM
Secondary Goat Anti-Mouse IgG H&L (Alexa Fluor^®^ 488)	ab150113	ABCAM

### Staining quantification

Images were analysed using FIJI, wherein images were masked for individual organoids and were quantified for fluorescence intensity signals for individual channels (DAPI, Alexa Fluor^®^ 488, Alexa Fluor^®^ 555) using the “Measure” function. The total intensity signal (Mean⋅Area) was normalised to the DAPI to produce a normalised relative fluorescence signal for each channel.

### Statistics

Statistics were calculated via Prism 10 & RStudio. Repeated measures one-way ANOVA was used for time series comparisons, followed by a multiple comparisons *post hoc* Tukey’s correction test. Student’s t-test was used for non-time series comparisons. A binomial test was used for categorical comparison of organoid set-up success. MANOVA was used to calculate the significance across the principal components. Code is attached on GitHub. https://github.com/monchablemelon/alternative_dissociation.

## Electronic supplementary material

Below is the link to the electronic supplementary material.

Supplementary Material 1

Supplementary Material 2

## Data Availability

Data is provided in the supplementary files.
