# Supplementary material for: Automated tissue dissociation for the establishment of human intestinal organoids
Source: Sci Rep. 2025 Jun 5;15:19813. doi: 10.1038/s41598-025-03905-9 (PMC12141665; doi:10.1038/s41598-025-03905-9)
Supplement: Supplementary file 2 — Supplementary Material 2 [file 41598_2025_3905_MOESM2_ESM.docx]

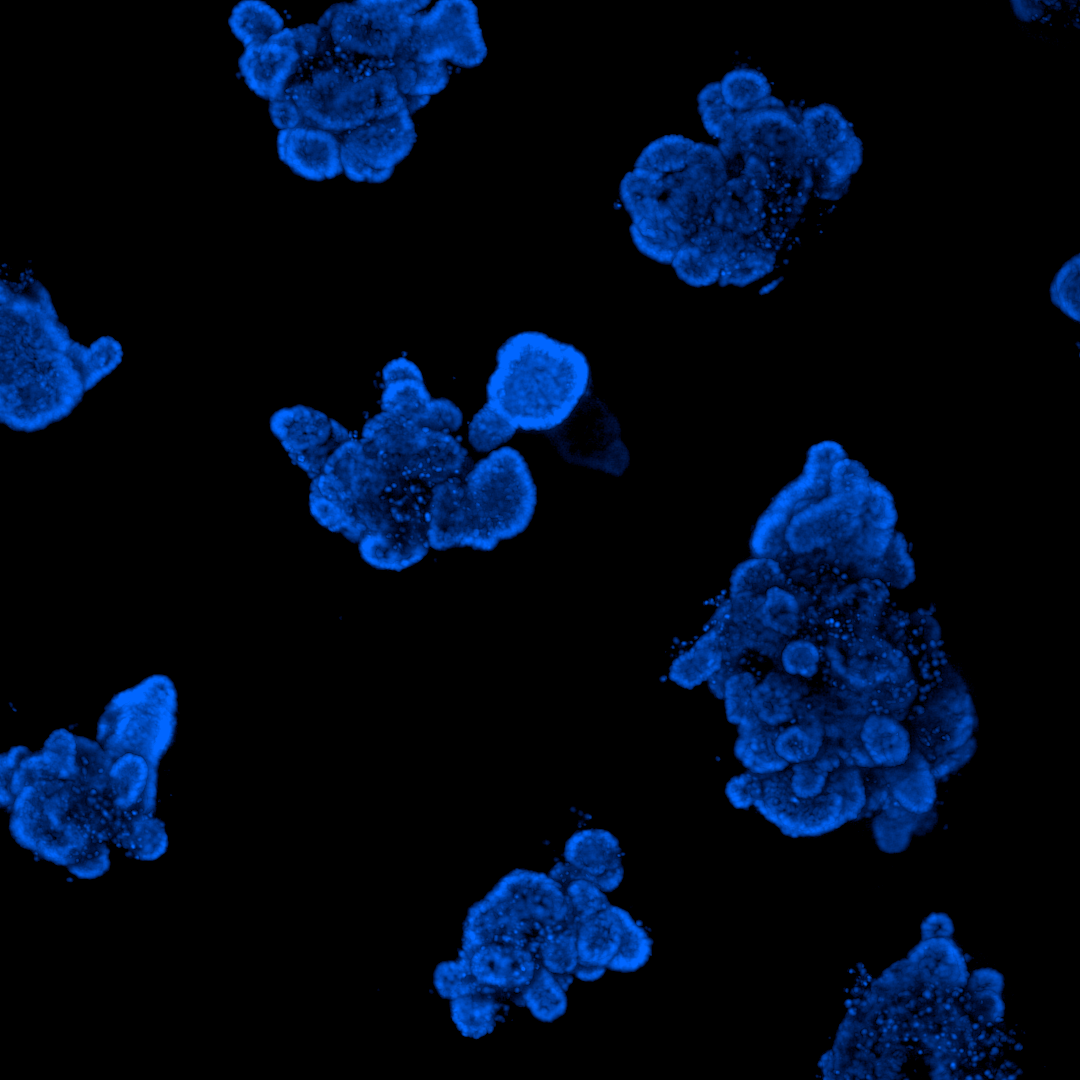

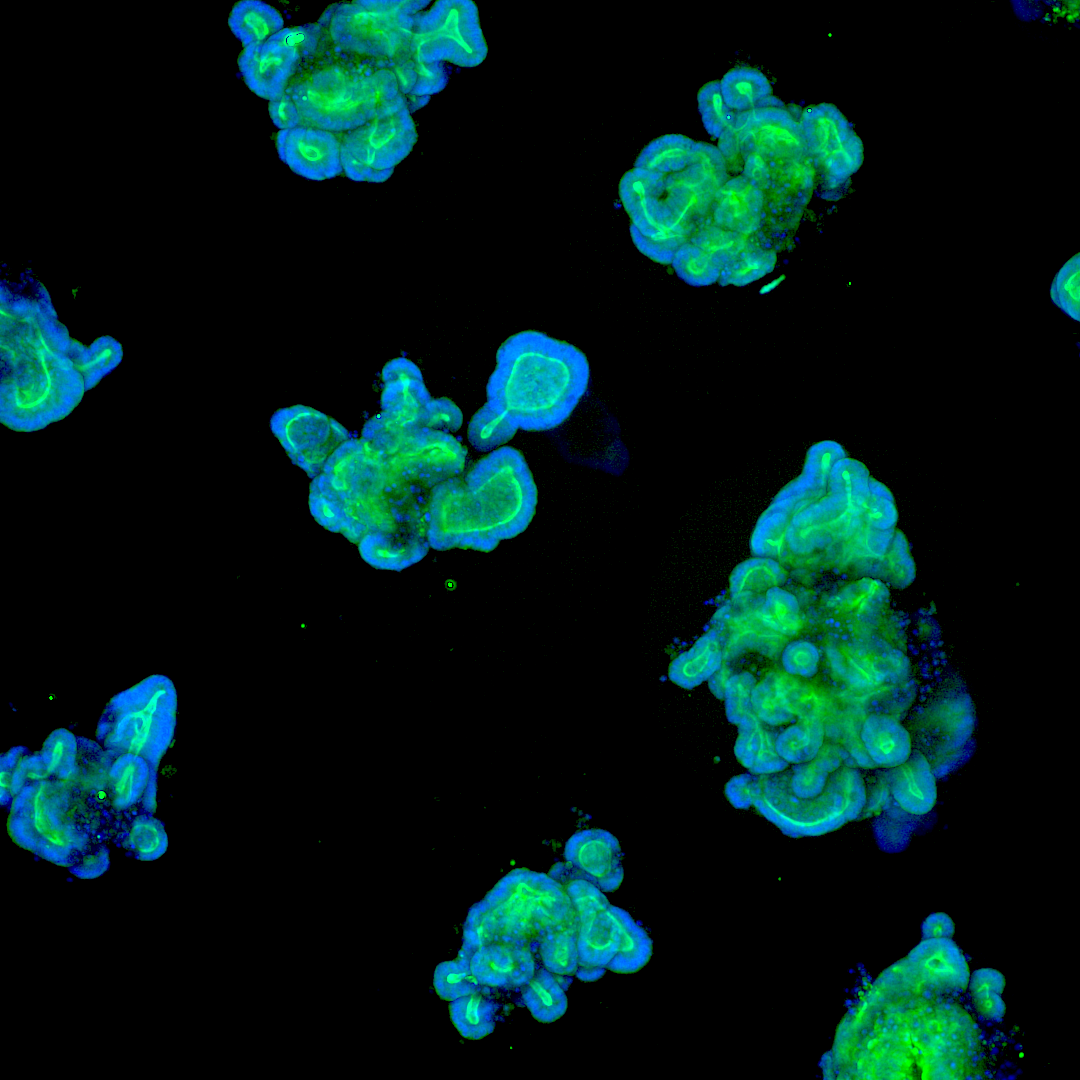

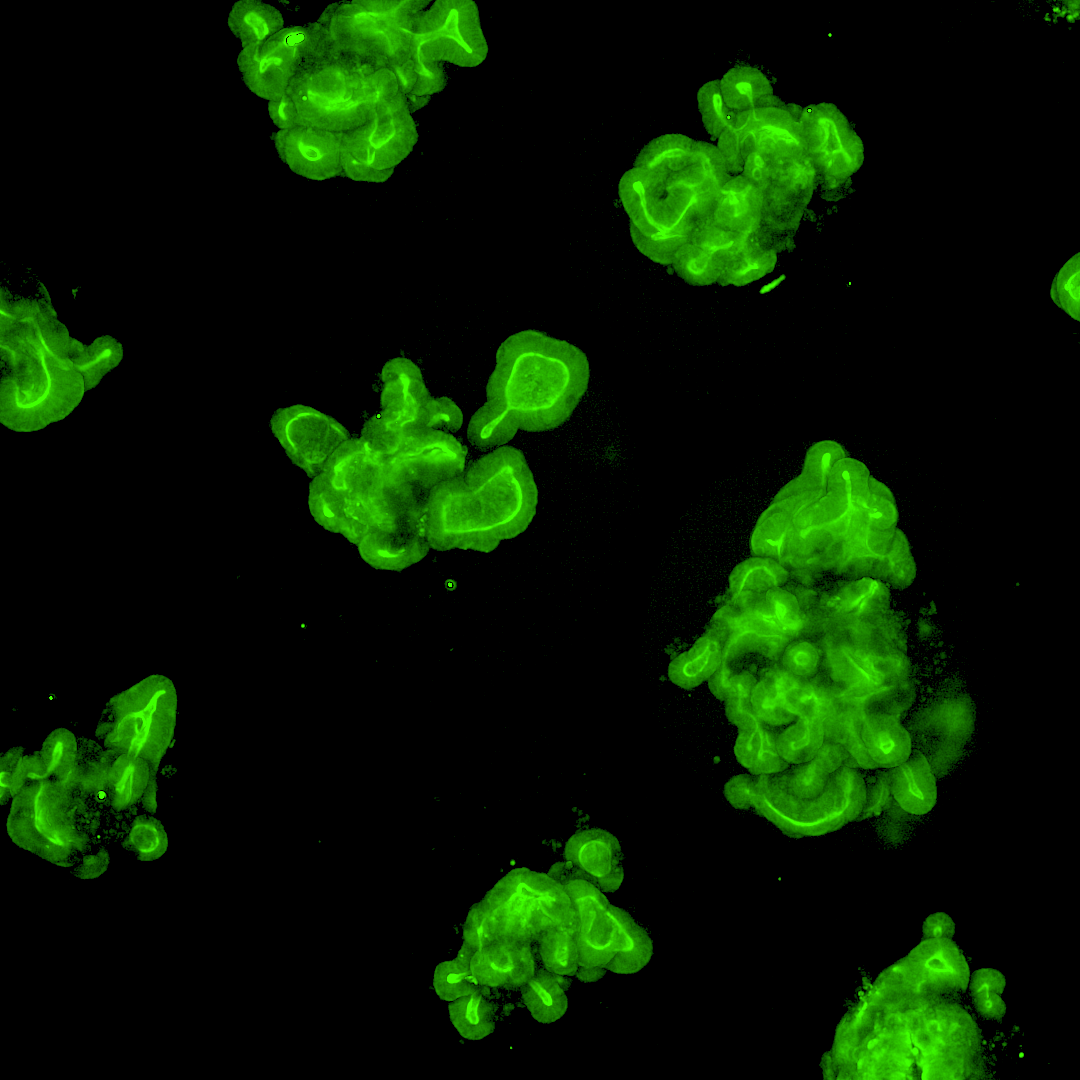


DAPI

Villin

Trans

Villin

Merge

Supplementary Figure 1) Small Intestinal organoids dissociated via the semi-automated pipeline stained with Villin and DAPI. Villin shows apical restriction. Scale bar 250 µm.

DAPI
